# Supplementary material for: Early Adverse Event Derived Biomarkers in Predicting Clinical Outcomes in Patients with Advanced Non-Small Cell Lung Cancer Treated with Immunotherapy
Source: Cancers (Basel). 2023 Apr 28;15(9):2521. doi: 10.3390/cancers15092521 (PMC10177532; doi:10.3390/cancers15092521)

Supplementary Table S1: Patient A in Figure 1 (Overall AE level)

AE-derived markers (Patient A for overall AE level)					
		Measurement type			
		Occurrence	Sum of all unique AEs	Sum of all AEs	Sum of all AE duration (days)
Grade/ Treatment relatedness	Any-grade	1	6	7	451
	Treatment related any grade	1	3	3	344
	Low-grade (1 or 2)	1	6	7	451
	Treatment related low-grade	1	3	3	344
	High-grade (3 or higher)	0	0	0	0
	Treatment related high-grade	0	0	0	0

Supplementary Table S2: Patient B in Figure 1 (Overall AE level)

AE-derived markers (Patient B for overall AE level)					
		Measurement type			
		Occurrence	Sum of all unique AEs	Sum of all AEs	Sum of all AE duration (days)
Grade/ Treatment relatedness	Any-grade	1	4	5	572
	Treatment related any-grade	1	1	1	169
	Low-grade (1 or 2)	1	3	4	568
	Treatment related low-grade	1	1	1	169
	High-grade (3 or higher)	1	1	1	4
	Treatment related high-grade	0	0	0	0

Supplementary Table S3: Patient A in Figure 1 (gastrointestinal disorders)

AE-derived biomarkers (Patient A for gastrointestinal disorders)					
		Measurement type			
		Occurrence	Sum of all unique AEs	Sum of all AEs	Sum of all AE duration (days)
Grade/ Treatment relatedness	Any grade	1	3	3	31
	Any treatment related grade	0	0	0	0
	Low-grade (1 or 2)	1	3	3	31
	Treatment related low-grade	0	0	0	0
	High-grade (3 or higher)	0	0	0	0
	Treatment related high-grade	0	0	0	0

Supplementary Table S4: Patient A in Figure 1 (weight loss)

AE-derived markers (Patient A for weight loss)					
		Measurement type			
		Occurrence	Sum of all unique AEs	Sum of all AEs	Sum of all AE duration (days)
Grade/ Treatment relatedness	Any-grade	1	1	2	168
	Treatment related any-grade	1	1	1	92
	Low-grade (1 or 2)	1	1	2	168
	Treatment related low-grade	1	1	1	92
	High-grade (3 or higher)	0	0	0	0
	Treatment related high-grade	0	0	0	0

Supplementary Table S5: Patient B in Figure 1 (Early AE)

Early AE-derived markers (Patient B)						
		Measurement type				
		Occurrence	Sum of all unique AEs	Sum of all AEs	Sum of all AE duration (days)
Grade/<br>Treatment relatedness	Any-grade	1	2	2	34
	Treatment related any-grade	0	0	0	0
	Low-grade (1 or 2)	1	1	1	30
	Treatment related low-grade	0	0	0	0
	High-grade (3 or higher)	1	1	1	4
	Treatment related high-grade	0	0	0	0

Supplementary Table S6: Summary of toxicity categories and individual early AEs associated with OS and PFS in Cohort A

**Toxicity category**	**AE**	**Improved OS**	**Poorer OS**	**Improved PFS**	**Poorer PFS**
*Trt: treatment related AE					
**Overall AE**		Any grade.Trt*<br>Low-Grade.Trt	High-Grade<br>High-Grade.Trt	Any grade.Trt<br>Low-Grade.Trt	High-Grade
**Blood and lymphatic system disorders**			High-Grade		
**Blood and lymphatic system disorders**	Anemia		High-Grade		
**Endocrine disorders**				Any grade<br>Any grade.Trt<br>Low-Grade<br>Low-Grade.Trt	
**Endocrine disorders**	Hypothyroidism			Any grade<br>Any grade.Trt<br>Low-Grade<br>Low-Grade.Trt	
**Eye disorders**				Any grade<br>Low-Grade	
**Gastrointestinal disorders**		Low-Grade	High-Grade<br>High-Grade.Trt		High-Grade
**Gastrointestinal disorders**	Diarrhea	Low-Grade	Any grade.Trt<br>High-Grade.Trt		
**Gastrointestinal disorders**	Vomiting		High-Grade<br>High-Grade.Trt		High-Grade
**General disorders and administration site conditions**			High-Grade		High-Grade
**Infections and infestations**			High-Grade		High-Grade
**Infections and infestations**	Lung infection				Any grade<br>High-Grade
**Investigations**	Alanine aminotransferase increased		Any grade<br>Any grade.Trt<br>Low-Grade<br>Low-Grade.Trt		
**Musculoskeletal and connective tissue disorders**			Any grade		Any grade

					High-Grade
**Musculoskeletal and connective tissue disorders**	Back pain		Any grade		Any grade High-Grade
**Psychiatric disorders**			Any grade Low-Grade		Any grade Low-Grade
**Psychiatric disorders**	Confusion		Any grade Low-Grade		Any grade Low-Grade
**Respiratory, thoracic and mediastinal disorders**			Any grade High-Grade		Any grade Low-Grade High-Grade
**Respiratory, thoracic and mediastinal disorders**	Aspiration		Any grade High-Grade		
**Respiratory, thoracic and mediastinal disorders**	Atelectasis				Any grade High-Grade
**Respiratory, thoracic and mediastinal disorders**	Cough				Any grade Low-Grade
**Respiratory, thoracic and mediastinal disorders**	Dyspnea				Any grade High-Grade
**Respiratory, thoracic and mediastinal disorders**	Pleural effusion				Any grade Low-Grade
**Respiratory, thoracic and mediastinal disorders**	Pneumothorax				Any grade Low-Grade
**Vascular disorders**			Any grade		Any grade High-Grade
**Vascular disorders**	Thromboembolic event				Any grade High-Grade

Supplementary Table S7: Summary of toxicity categories and individual early AEs associated with treatment response in Cohort A

**Toxicity category**	**AE**	**PD Association**	**DC Association**
Trt: treatment related AE			
**Overall AE**		High-Grade	Any grade.Trt<br>Low-Grade<br>Low-Grade.Trt
**General disorders and administration site conditions**			Low-Grade
**General disorders and administration site conditions**	Fatigue		Any grade<br>Low-Grade
**General disorders and administration site conditions**	Pain		Low-Grade
**Investigations**	Platelet count decreased		Any grade<br>Any grade.Trt<br>Low-Grade<br>Low-Grade.Trt

Supplementary Table S8: Summary of toxicity categories and individual early AEs associated with duration of treatment (DOT) in Cohort A

**Toxicity category**	**AE**	**Negative Correlation**	**Positive Correlation**
Trt: treatment related AE			
**Overall AE**		High-Grade	
**Endocrine disorders**			Any grade<br>Any grade.Trt<br>Low-Grade<br>Low-Grade.Trt
**Endocrine disorders**	Hypothyroidism		Any grade<br>Any grade.Trt<br>Low-Grade<br>Low-Grade.Trt
**Eye disorders**			Any grade<br>Low-Grade
**Eye disorders**	Eye disorders - Other, specify		Any grade<br>Low-Grade
**Gastrointestinal disorders**	Dry mouth		Any grade<br>Low-Grade
**Gastrointestinal disorders**	Vomiting		Any grade<br>Any grade.Trt<br>Low-Grade<br>Low-Grade.Trt
**Nervous system disorders**	Dizziness		Any grade<br>Low-Grade
**Nervous system disorders**	Headache		Any grade<br>Low-Grade
**Skin and subcutaneous tissue disorders**			Any grade<br>Low-Grade
**Skin and subcutaneous tissue disorders**	Dry skin		Any grade<br>Low-Grade

Supplementary Table S9: Summary of toxicity categories and individual early AEs associated with OS and PFS in Cohort B

Toxicity category	AE	Improved OS	Poorer OS	Improved PFS	Poorer PFS
Trt: treatment related AE					
**Overall AE**		Low-Grade	High-Grade	Low-Grade	High-Grade
**Gastrointestinal disorders**		Low-Grade	High-Grade	Any grade<br>Low-Grade	High-Grade
**Gastrointestinal disorders**	Abdominal pain		Any grade<br>High-Grade		Any grade<br>High-Grade
**Gastrointestinal disorders**	Dry mouth		Any grade<br>Low-Grade		
**Gastrointestinal disorders**	Dysphagia		Any grade<br>Low-Grade<br>High-Grade		Any grade
**Gastrointestinal disorders**	Nausea			Any grade<br>Low-Grade	
**General disorders and administration site conditions**			Any grade<br>High-Grade		
**General disorders and administration site conditions**	Death NOS		Any grade<br>High-Grade		Any grade<br>High-Grade
**General disorders and administration site conditions**	Fatigue		High-Grade		High-Grade
**General disorders and administration site conditions**	Pain		Any grade<br>Low-Grade		
**Investigations**	Aspartate aminotransferase increased		High-Grade		
**Metabolism and nutrition disorders**			Any grade<br>Low-Grade		Any grade
**Metabolism and nutrition disorders**	Anorexia		Any grade<br>Low-Grade		
**Metabolism and nutrition disorders**	Hypoalbuminemia		Any grade<br>Low-Grade		Any grade<br>Low-Grade
**Musculoskeletal and connective tissue disorders**	Back pain		Any grade<br>Low-Grade		
**Psychiatric disorders**			Any grade<br>Low-Grade		Any grade<br>Low-Grade
**Psychiatric disorders**	Depression		Any grade<br>Low-Grade		Any grade<br>Low-Grade
**Respiratory, thoracic and mediastinal disorders**			Any grade<br>Low-Grade<br>High-Grade		Any grade<br>Low-Grade<br>High-Grade

**Respiratory, thoracic and mediastinal disorders**	Cough		Any grade<br>Low-Grade		Any grade<br>Low-Grade
**Respiratory, thoracic and mediastinal disorders**	Dyspnea		Any grade<br>Low-Grade		Any grade<br>Low-Grade
**Respiratory, thoracic and mediastinal disorders**	Pleural effusion		Any grade<br>High-Grade		Any grade<br>High-Grade
**Vascular disorders**			Low-Grade		Low-Grade
**Vascular disorders**	Hypertension		Any grade<br>Low-Grade		Any grade<br>Low-Grade

Supplementary Table S10: Summary of toxicity categories and individual early AEs associated with treatment response in Cohort B

**Toxicity category**	**AE**	**PD Association**	**DC Association**
Trt: treatment related AE			
**Overall AE**			Any grade.Trt<br>Low-Grade.Trt
**Gastrointestinal disorders**			Any grade<br>Any grade.Trt<br>Low-Grade<br>Low-Grade.Trt
**Gastrointestinal disorders**	Nausea		Any grade<br>Any grade.Trt<br>Low-Grade<br>Low-Grade.Trt

Supplementary Table S11: Summary of toxicity categories and individual early AEs associated with duration of treatment (DOT) in Cohort B

Toxicity category	AE	Negative.Correlation	Positive.Correlation
Trt: treatment related AE			
**Blood and lymphatic system disorders**	Anemia		Any grade<br>Low-Grade
**Gastrointestinal disorders**	Constipation		Any grade<br>Low-Grade
**Gastrointestinal disorders**	Flatulence		Any grade<br>Low-Grade
**Investigations**	Investigations - Other, specify		Any grade<br>Low-Grade
**Skin and subcutaneous tissue disorders**			Any grade<br>Low-Grade
**Skin and subcutaneous tissue disorders**	Rash maculo-papular		Any grade<br>Low-Grade

Supplementary Figure S1: Patient A without early AE event

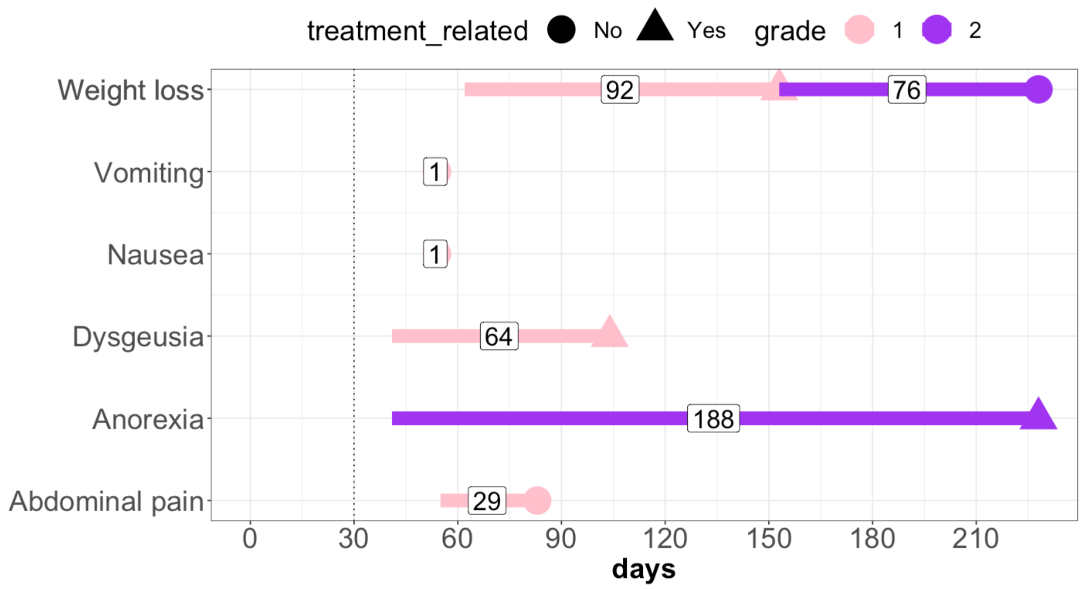

Supplementary Figure S2: Patient B with early AE event

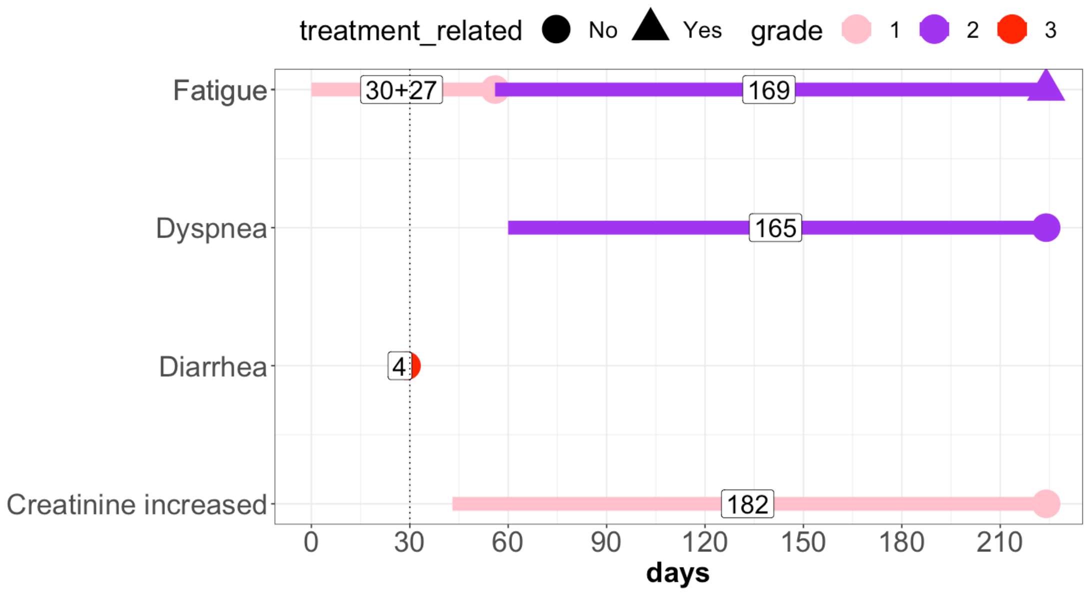

Supplementary Figure S3: Association of high-grade early AE-derived biomarkers with poor survival outcomes in cohort A

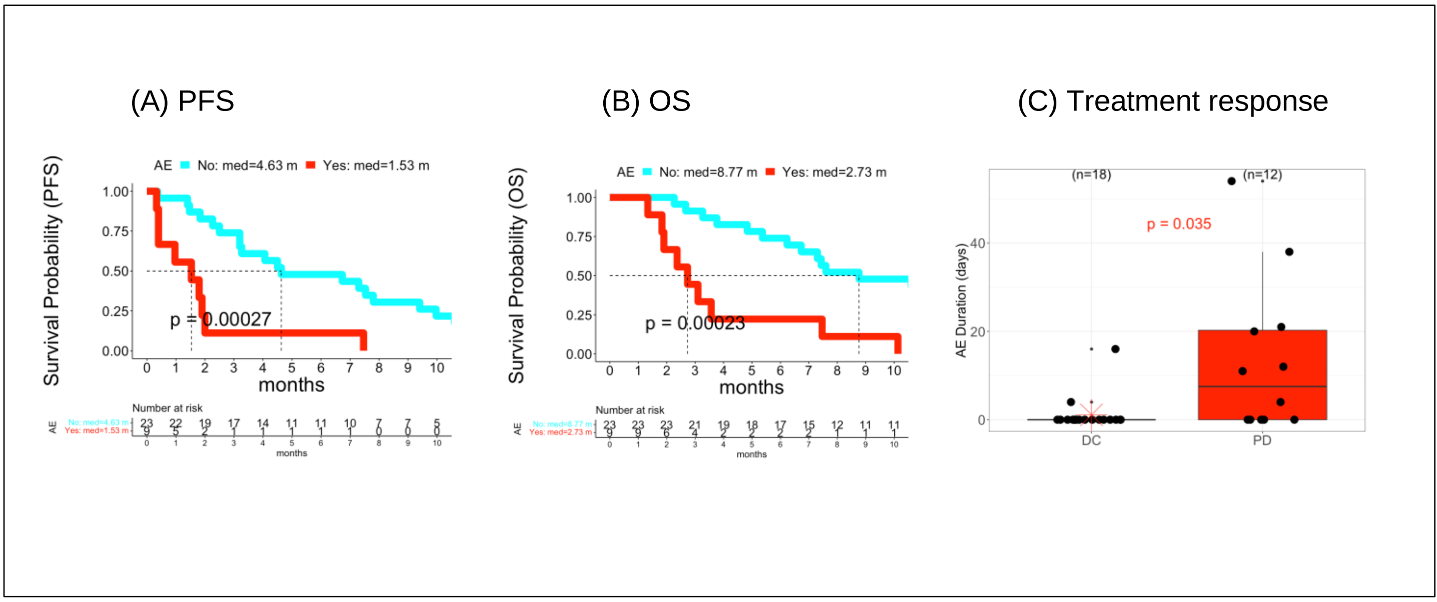

Supplement: Supplementary file 1 [file cancers-15-02521-s001.zip › cancers-2303980-supplementary.pdf]
